# Light-Exposed Metabolic Responses of *Cordyceps militaris* through Transcriptome-Integrated Genome-Scale Modeling

**DOI:** 10.3390/biology13030139

**Published:** 2024-02-22

**Authors:** Panyawarin Soommat, Nachon Raethong, Ratchaprapa Ruengsang, Roypim Thananusak, Teeraphan Laomettachit, Kobkul Laoteng, Treenut Saithong, Wanwipa Vongsangnak

**Affiliations:** 1 Genetic Engineering and Bioinformatics Program, Graduate School, Kasetsart University, Bangkok 10900, Thailand; panyawarin.so@ku.th; 2 Institute of Nutrition, Mahidol University, Nakhon Pathom 73170, Thailand; nachon.rae@mahidol.ac.th; 3 Bioinformatics and Systems Biology Program, School of Bioresources and Technology and School of Information Technology, King Mongkut’s University of Technology Thonburi (Bang Khun Thian), Bangkok 10150, Thailand; ratchaprapa.kams@kmutt.ac.th (R.R.); teeraphan.lao@kmutt.ac.th (T.L.); 4 Omics Center for Agriculture, Bioresources, Food, and Health, Kasetsart University (OmiKU), Bangkok 10900, Thailand; roypim.tha@ku.th; 5 Industrial Bioprocess Technology Research Team, Functional Ingredients and Food Innovation Research Group, National Center for Genetic Engineering and Biotechnology (BIOTEC), National Science and Technology Development Agency (NSTDA), Pathum Thani 12120, Thailand; kobkul@biotec.or.th; 6 Center for Agricultural Systems Biology (CASB), Systems Biology and Bioinformatics Research Group, Pilot Plant Development and Training Institute, King Mongkut’s University of Technology Thonburi (Bang Khun Thian), Bangkok 10150, Thailand; 7 Department of Zoology, Faculty of Science, Kasetsart University, Bangkok 10900, Thailand

**Keywords:** *Cordyceps militaris*, light, metabolism, transcriptome-integrated GSMM (*ti*GSMM), systems biology

## Abstract

**Simple Summary:**

*Cordyceps militaris* is an entomopathogenic fungus with potential health benefits. These benefits have made this fungus marketable. Employing the transcriptome-integrated genome-scale metabolic model approach using gene inactivity moderated by metabolism and expression framework (*i*PS1474-*ti*GSMM), this work reveals metabolic fluxes in correlation with the expressed genes involved in the cordycepin and carotenoid biosynthetic pathways of *C. militaris* under light exposure. Additionally, an analysis of reporter metabolites emphasizes central carbon, purine, and fatty acid metabolisms, uncovering crucial processes for *C. militaris* adaptation to light exposure.

**Abstract:**

The genome-scale metabolic model (GSMM) of *Cordyceps militaris* provides a comprehensive basis of carbon assimilation for cell growth and metabolite production. However, the model with a simple mass balance concept shows limited capability to probe the metabolic responses of *C. militaris* under light exposure. This study, therefore, employed the transcriptome-integrated GSMM approach to extend the investigation of *C. militaris*’s metabolism under light conditions. Through the gene inactivity moderated by metabolism and expression (GIMME) framework, the *i*PS1474-*ti*GSMM model was furnished with the transcriptome data, thus providing a simulation that described reasonably well the metabolic responses underlying the phenotypic observation of *C. militaris* under the particular light conditions. The *i*PS1474-*ti*GSMM obviously showed an improved prediction of metabolic fluxes in correlation with the expressed genes involved in the cordycepin and carotenoid biosynthetic pathways under the sucrose culturing conditions. Further analysis of reporter metabolites suggested that the central carbon, purine, and fatty acid metabolisms towards carotenoid biosynthesis were the predominant metabolic processes responsible in light conditions. This finding highlights the key responsive processes enabling the acclimatization of *C. militaris* metabolism in varying light conditions. This study provides a valuable perspective on manipulating metabolic genes and fluxes towards the target metabolite production of *C. militaris*.

## 1. Introduction

*Cordyceps militaris* is an entomopathogenic fungus belonging to the class Ascomycota which grows on the larva or pupa of lepidoptera insects. It has been used in traditional medicine in China and east Asia [1]. *C. militaris* contains several active components, such as cordycepin (3′-deoxyadenosine), adenosine, mannitol, polysaccharide, ergosterol, and carotenoid [2,3]. These substances possess biological activities, including anti-oxidant, anti-fungal, anti-bacterial, and immunomodulatory effects and anti-inflammatory activities [4,5,6,7,8,9,10].

These health benefits have made this fungus marketable. Thus, the optimal cultivation process for improving the cell growth and production yield of the target metabolites of *C. militaris* has become an active area of biotechnological research and development [3]. A previous study demonstrated that the contents of bioactive compounds in *C. militaris*, especially cordycepin, were altered in response to light conditions [11]. Short-wavelength light significantly increased the total carotenoid content in fruiting bodies of *C. militaris* [12]. The recent study of Thananusak et al. (2020) [13] also showed light conditions affecting the physiology of *C. militaris*, in which the response mechanism was proposed. The metabolism of *C. militaris* has been previously explored by using genome-scale metabolic models (GSMM), e.g., *i*NR1329 [14] and *i*PC1469 [15]. They revealed sugar utilization for growth and lipid biosynthetic capability under dark conditions, respectively. However, FBA models often weakly predict metabolic fluxes due to minimal data requirements and simplicity. Additionally, the context-specific metabolic behavior underlying *C. militaris* growth and metabolite production under specific light exposure remains largely unknown. Investigating the metabolic responses of *C. militaris* under light exposure is limited.

To address this challenge, transcriptome data were integrated into GSMMs to represent cellular functionalities by extracting a subset of reactions in relation to a particular context, e.g., a specific condition. Several integrative approaches have been developed based on the rationales, including GIMME [16], iMAT [17], and RegrEx [18]. Among these, GIMME stands out as the simplest method with minimal information requirements. The GIMME (Gene Inactivity Moderated by Metabolism and Expression) approach minimizes the flux of reaction with lowly expressed genes through the binarization of enzyme abundance levels to “ON” or “OFF” states after thresholding the associated gene expression level [16]. The approach has been effectively utilized across various organisms, including microorganisms like *E. coli* [16] and Methanothermobacter [19], as well as plants such as Arabidopsis [20] and cassava [21].

This study, therefore, aimed to employ the transcriptome-integrated GSMM approach to further investigate the metabolic responses of *C. militaris* to light conditions. Initially, the GSMM of *C. militaris* was retrofitted for growth under light conditions. Next, transcriptome data under light exposure were analyzed and further used to reconstruct the GSMM with transcriptome-based constraints using the GIMME algorithm [16]. To uncover the key metabolites underlying the identified potential metabolic routes, the *i*PS1474-*ti*GSMM of *C. militaris* was also used. This study offers an effective approach to investigating cellular metabolism, particularly the key metabolic processes and the involved components acting to achieve the desirable condition.

## 2. Materials and Methods

### 2.1. Retrofitting GSMM of C. militaris for Growth under Light Conditions

The GSMMs of *i*NR1329 [14] and *i*PC1469 [15] were initially used as scaffolds to retrofit the GSMM of *C. militaris*, then called *i*PS1474. A combination of various information types was essential to carry out a retrofitting metabolic network. Essential information was collected from the enhanced annotation data of *C. militaris* protein sequences, biochemical pathways, and chemical identifiers underlying the KEGG [22], MetaCyc [23] databases, and RAVEN toolbox 2.0 [24], publications on specific enzymes, protein databases, and also the literature [13]. In addition, there was physiological evidence for the presence of a reaction or a pathway in *C. militaris*, e.g., a biosynthetic pathway of carotenoid was also added. The biomass composition reaction of GSMM was also retrofitted for carotenoid biosynthesis [13]. In the processes of stoichiometry for cofactors, as well as the information on the reversibility or irreversibility for each reaction, these were added as information into the network. The identification of sub-cellular localization was additionally considered. All scripts for retrofitting the GSMM of *C. militaris*, as well as the generated model files (.mat, .xml, .xlsx, .txt,), were deposited into a public repository on GitHub (https://github.com/sysbiomics/Cordyceps_militaris-tiGSMM) on 26 January 2024.

### 2.2. Validation of the Retrofitted GSMM of C. militaris

The constraint-based flux simulation of *i*PS1474 was performed using a flux balance analysis (FBA) in RAVEN 2.0 [24], MATLAB (R2020b) [25], and the Gurobi optimizer as the linear programming solver. The objective function was set to maximize cell growth under carbon uptake rates based on experimental data. The uptake rate for glucose or sucrose under light exposure conditions was set to be 0.1593 or 0.0845 mmol gDW^−1^ h^−1^, respectively [13]. The exchange fluxes of ammonium, phosphate, sulfate, H_2_O, and H^+^ were unconstrained to provide the basic nutrients for cell growth.

The *i*PS1474 model was validated with in vitro data [13] for growth and biomass under different carbon sources, i.e., glucose or sucrose, upon light exposure. Additionally, constraints on maximizing cordycepin and carotenoid production were imposed to investigate metabolic capability throughout this study. The metabolic conversion of *C. militaris* was iteratively tuned with the gene expression data of *C. militaris* under light exposure.

### 2.3. Transcriptome-Integrated Constraint-Based Metabolic Model of C. militaris Using GIMME

Initially, the RNA-seq datasets of *C. militaris* under light conditions were retrieved from a previous study [13]. The transcriptome data, including the expression of 8747 genes, were normalized based on the fragments per kilobase of the transcript per million mapped reads (FPKM) (Supplementary File S1; Table S1). Of these, 1412 expressed genes associated with metabolic reactions were integrated into the *i*PS1474 model through a gene inactivity moderated by metabolism and expression analysis (GIMME) algorithm, resulting in the transcriptome-integrated model, hereafter called *i*PS1474-*ti*GSMM. Briefly, GIMME identifies and eliminates inactive reactions associated with genes expressing as a defined threshold and reintegrates reactions essential for achieving the objective function, ensuring that their expression meets [16]. At first, the algorithm determined the maximum achievable flux through the objective function, i.e., the growth rate, and employed the flux value as the boundary for reactions. Then, the algorithm identified active reactions by minimizing the influence of inactive reactions based on gene expression-weight coefficients by linear programming according to Equation (1).

Minimize: ∑*_i_*
*c_i_* · |*v_i_*|. Subject to: *S_ij_* · *v_i_* = 0, lb_i_ < *v_i_* < ub_i_, *c_i_* = {*x*_threshold_ − *x_i_* where *x*_threshold_ > *x_i_*, 0 otherwise}, (1)

It is noted that *S_ij_* is a stoichiometric coefficient of *j* metabolites and *i* reactions; *v_i_* is a flux flow through reaction *i*; lb_i_ and ub_i_ are the lower and upper bounds of the flux through reaction *i*; *x_i_* is normalized gene expression data mapped to reaction *i*; *x*_threshold_ is the gene expression level threshold; and *c_i_* is the penalty score.

The gene expression level threshold was set at the 50th percentile of all possible gene expressions in *i*PS1474-*ti*GSMM of *C. militaris*. For the simulation, it was conducted in COBRA Toolbox version 3.0 with a glpk solver on MATLAB (R2020b).

### 2.4. Analysis of Active Flux Distribution and Metabolic Reaction under Light Exposure

The concurrence of the flux prediction and transcriptome data was assessed for metabolic flux distribution under light exposure. The *i*PS1474-*ti*GSMM simulations of *C. militaris* under different carbon sources, i.e., glucose or sucrose, were carried out. The predicted metabolic fluxes were compared with the metabolic gene expression. The active light-exposed metabolic reactions (non-zero fluxes) and inactive (zero-fluxes) reactions were integrated based on the presence and absence of gene expression, respectively.

### 2.5. Using the Retrofitted GSMM for Identifying Reporter Metabolites and Key Sub-Networks

To identify reporter metabolites and key sub-networks in responses to light exposure, the retrofitted GSMM was used. We applied the reporter metabolite algorithm to identify reporter metabolites and search for highly correlated metabolic sub-networks for pairwise light/dark comparison. This analysis used gene-level statistics (e.g., *p*-value), a set of genes, and their associated metabolites as the inputs. If a metabolite had a distinct-directional *p*-value below 0.05 (pDistinctDirUp or pDistinctDirDn in the R package Piano) [26], it was identified as a significant reporter metabolite. In a similar manner, a set of genes and their corresponding reactions associated with reporter metabolites was considered as a significant metabolic sub-network.

## 3. Results and Discussion

### 3.1. The Retrofitted iPS1474-GSMM of C. militaris in Comparison with Earlier Model Characteristics

**Figure 1:**
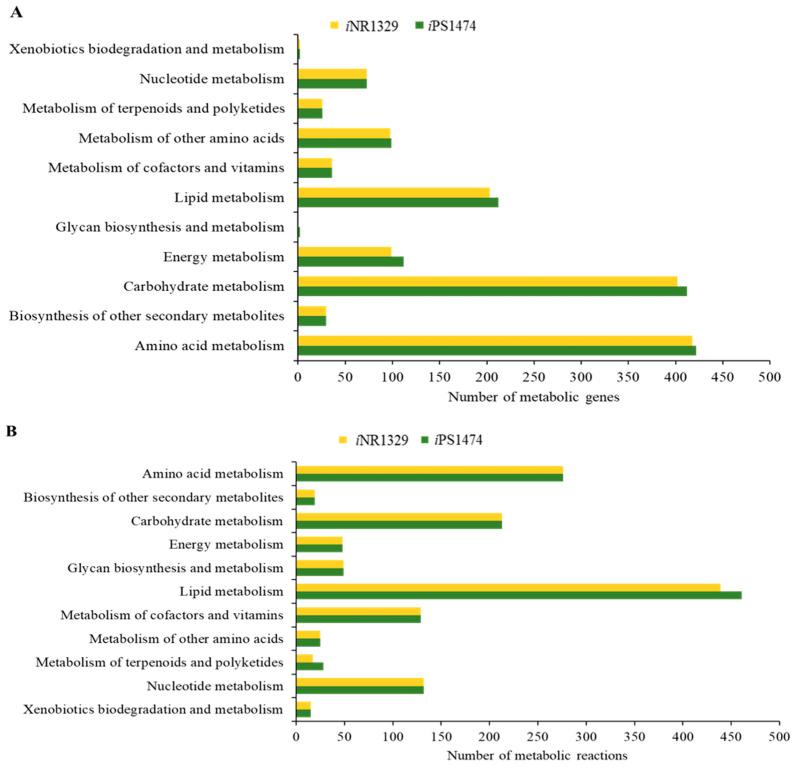
(**A**) Number of metabolic genes and (**B**) number of metabolic reactions in comparison between *i*NR1329 and *i*PS1474 of *C. militaris*.

Functional assignment and enhanced network reconstruction using bioinformatics resulted in a retrofitted GSMM (*i*PS1474 model) of *C. militaris*, as shown in Table 1. This contained 1474 genes and 1916 metabolic reactions that governed 1245 metabolites amongst four compartments, i.e., cytosol, mitochondria, peroxisome, and extracellular space. Compared with the earlier model, *i*NR1329, created by Raethong et al., 2020 [14], this result indicates that 145 metabolic genes were uniquely identified in *i*PS1474, which were mainly identified in primary metabolic processes, including amino acid metabolism, carbohydrate metabolism, energy metabolism, lipid metabolism, and glycan biosynthesis and metabolism (Figure 1A). Moreover, 96 unique metabolic reactions with 12 unique EC numbers were mostly involved in lipid metabolism and the metabolism of terpenoids and polyketides, as shown in Figure 1B. Overall, the metabolic reactions for the *i*PS1474 model were particularly enriched in the lipid, carbohydrate, and amino acid metabolisms.

**Figure 2:**
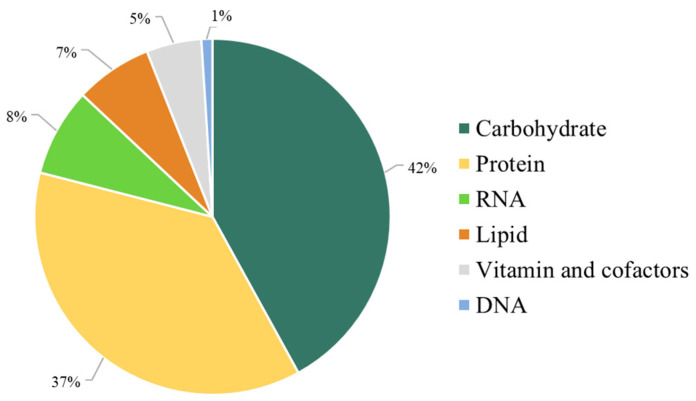
The *i*PS1474 metabolic features in the context of biomass composition.

Moreover, the stoichiometry of the metabolic precursors involved in the biomass composition was quantitatively estimated based on the macromolecular contents of carbohydrates, proteins, lipids, nucleotides, and vitamins of *C. militaris* obtained from research supports (Figure 2).

### 3.2. Validation and Performance of iPS1474-GSMM Model

**Figure 3:**
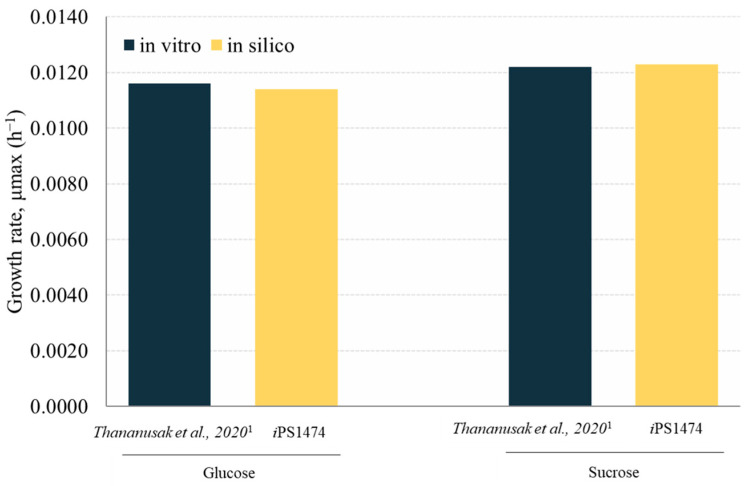
Validation of *i*PS1474 by comparison of growth rate (h^−1^) between in silico and in vitro data across different carbon sources, i.e., glucose and sucrose. Note: Data were taken from ^1^ Thananusak et al., 2020 [26].

The retrofitted GSMM (*i*PS1474 model) was validated based on the study of Thananusak et al. (2020) [13]. The model simulated a capability for fungal cell growth in glucose and sucrose cultures under light exposure against measured data from laboratories [13]. By constraining the uptake rates of individual carbon sources, *i*PS1474 well predicted the growth of *C. militaris* in varied carbon substrates (error percentage ≤ 1.64, Figure 3). This result indicates the predictive performance of the *i*PS1474 model for simulating fungal cell growth in these carbon sources.

### 3.3. Transcriptome-Integrated GSMM (iPS1474-tiGSMM) Model of C. militaris under Light Conditions

The *i*PS1474 model well simulated the growth rate of *C. militaris* in accordance with the experimental data. However, as fluxes dynamically change in response to varying environments, integrating the model with transcriptome data under light conditions became essential to capture the activity of a specific subset of reactions. The expression of enzyme-encoding genes is a basic clue indicating the activity of biochemical reactions in action under a particular condition. Assuming all enzymes functioned independently, a metabolic reaction was proposed to activate at least one related expressed gene. According to Thananusak et al. (2020) [13], the 8747 genes of *C. militaris* were expressed in glucose or sucrose cultures under light conditions, allowing *i*PS1474 to initially activate reactions related to 1412 expressed metabolic genes.

**Figure 4:**
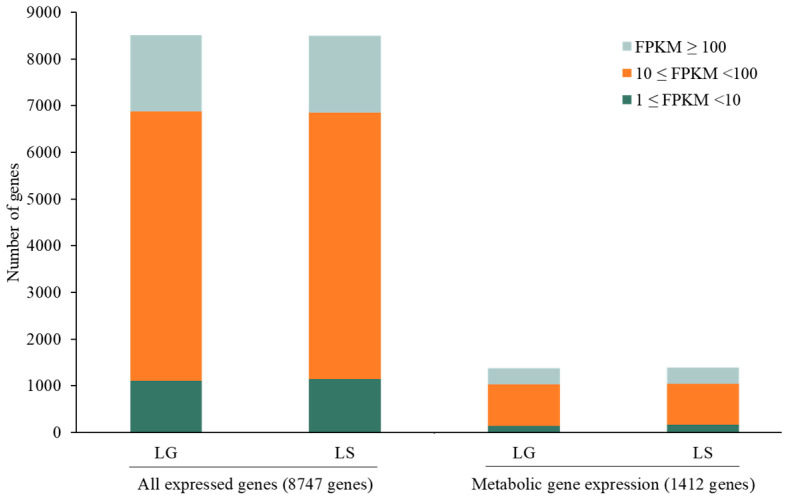
Bar plot shows distributing gene expression levels across different FPKM values of all expressed genes and metabolic genes in light–glucose (LG) and light–sucrose (LS) conditions.

In detail, 93.62% and 94.17% of the 8747 genes were expressed in light–glucose (LG) and light–sucrose (LS) conditions, respectively. The expression levels of all genes and metabolic genes were classified into three categories. The majority of genes with FPKM values were 10 ≤ FPKM < 100, FPKM ≥ 100, and 1 ≤ FPKM < 10, respectively. The total expressed metabolic genes in each category are shown in Figure 4.

**Figure 5:**
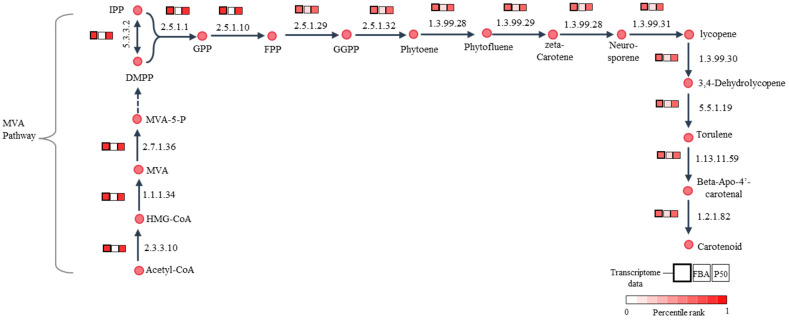
Illustration of comparative percentile rank between transcriptome data of *C. militaris* under sucrose upon light exposure [13], the predicted fluxes of *i*PS1474 (FBA) and *i*PS1474-*ti*GSMM-P50 for carotenoid biosynthetic pathway. Abbreviated metabolite names are as follows: HMG-CoA, 3-hydroxy-3-methylglutaryl-CoA; MVA, mevalonate; MVA-5-P, 5-phosphomevalonate; IPP, isopentenyl diphosphate; DMPP, dimethylallyl diphosphate; GPP, geranyl diphosphate; FPP, farnesyl diphosphate; and GGPP, Geranylgeranyl diphosphate.

Here, GIMME utilized 1412 expressed metabolic genes to inform the set of active reactions in the *i*PS1474 model by assuming that reactions were considered to be active when the expression of associated genes exceeded a certain threshold (50th percentile, P50) and minimized flux through inactive reactions. As a result, the GSMM with transcriptome-integrated constraints (*ti*GSMM) of *C. militaris*, namely, *i*PS1474-*ti*GSMM-P50, was gained using the GIMME approach. The simulation of *i*PS1474-*ti*GSMM-P50 under sucrose upon light exposure was assessed for its capability to predict flux distribution consistent with the transcriptome data of *C. militaris* (Supplementary File S2; Table S2). The *i*PS1474-*ti*GSMM-P50 showed flux distribution, which improved predicted flux through carotenoid biosynthesis via the mevalonate (MVA) pathway, as shown in Figure 5. It is suggested that the hydroxymethylglutaryl-CoA synthase (EC: 2.3.3.10) catalyzing 3-hydroxy-3-methylglutaryl-CoA (HMG-CoA) synthesis, hydroxymethylglutaryl-CoA reductase (EC:1.1.1.34) catalyzing mevalonate (MVA) synthesis, and mevalonate kinase (EC:2.7.1.36) catalyzing 5-Phosphomevalonate (MVA-5-P) synthesis as a substrate played crucial roles in the biosynthesis of carotenoid, as supported by Thananusak et al. (2020) [13]. Meanwhile, the *i*PS1474 model could not capture the MVA pathway (Figure 5). This finding clearly indicates that *i*PS1474-*ti*GSMM-P50 could improve the predicted metabolic flux distribution consistent with the light-exposed gene expression of *C. militaris*.

### 3.4. Active Light-Exposed Metabolic Reactions Predicted by iPS1474-tiGSMM-P50

**Figure 6:**
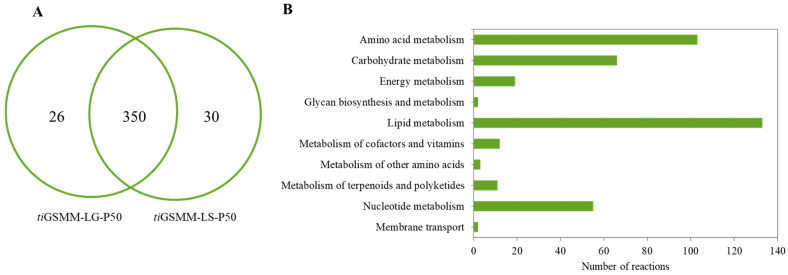
The number of the active metabolic reactions identified by the *i*PS1474-*ti*GSMM-P50 model prediction, (**A**) Venn diagram of the number of active reactions under glucose and sucrose upon light-exposure, i.e., *ti*GSMM-LG-P50 and *ti*GSMM-LS-P50, respectively, and (**B**) KEGG-based pathway classification of combination of all active reactions under light-exposure.

To investigate the light-exposed metabolic responses, *i*PS1474-*ti*GSMM-P50 simulations under glucose and sucrose upon light exposure were performed. The pie chart shows the number of active light-exposed metabolic reactions in glucose (376 reactions) and sucrose (380 reactions), as shown in Figure 6A. According to the combination of all the active reactions, 406 active light-exposed metabolic reactions were identified (Supplementary File S3; Table S3). These metabolic reactions were classified into 10 sub-categories involved in lipid metabolism (133 reactions), amino acid metabolism (103 reactions), carbohydrate metabolism (66 reactions), nucleotide metabolism (55 reactions), energy metabolism (19 reactions), the metabolism of cofactors and vitamins (12 reactions), the metabolism of terpenoids and polyketides (11 reactions), the metabolism of other amino acids (3 reactions), glycan biosynthesis and metabolism (2 reactions), and membrane transport (2 reactions), as shown in Figure 6B.

The characterization of the active metabolic reactions of *C. militaris* under light-exposure-induced responses should be further elucidated to dissect the impact of light on its metabolism and identify potential targets for enhancing the production of bioactive compounds. It has been reported that *C. militaris* could use a wide range of carbon sources [27]. Focusing on core metabolic reactions (350 reactions) (Figure 6A), interestingly, the important reactions involving the central metabolic pathways were active, such as glycolysis, the pentose-phosphate pathway, and the tricarboxylic acid (TCA) cycle. In glycolysis, r0005 associated with CCM_08316, encoding glucokinase (EC: 2.7.1.2), facilitates the phosphorylation of glucose to glucose-6-phosphate. It is involved in the first step of glycolysis, which is often regulated in response to cellular energy demands.

The reactions involved in the pentose phosphate pathway, e.g., R01056_c associated with CCM_00462, CCM_01641, and CCM_08256 (EC: 5.3.1.6), encoding for ribose-5-phosphate isomerase to generate ribose 5-phosphate, which is an intermediate metabolite for the synthesis of PRPP (5-phospho-alpha-ribose-1-diphosphate), were associated with CCM_02500, CCM_00434, and CCM_04665, encoding for ribose-phosphate diphosphokinase (EC: 2.7.6.1; r0336) involved in PRPP synthesis. It is an important precursor molecule involved in the biosynthesis of cordycepin [28].

Moreover, r48, associated with CCM_01079 and CCM_08657, encoding for pyruvate dehydrogenase (EC: 1.2.4.1), as well as CCM_05680 encoding for dihydrolipoyllysine-residue acetyltransferase (EC: 2.3.1.12; r0023), participated in the TCA cycle by converting pyruvate into acetyl-CoA.

Additionally, the reactions associated with carotenoid biosynthesis, such as r1143 (EC: 2.5.1.1) and r1144 (EC: 2.5.1.10), associated with the CCM_03203 gene encoding for farnesyl diphosphate (FPP) synthetase, were eventually responsible for enhanced carotenoid production, and rt_R0009 associated with the CCM_09155 gene encoding 4′-apo-beta carotenal oxygenase (EC: 1.2.1.82) was involved in carotenoid biosynthesis. Moreover, several reactions in cordycepin biosynthesis were identified, such as cordycepin (CCM_04436 and CCM_04437) and the 3 amp (CCM_04438) gene encoding for the cordycepin synthetase complex.

To gain deeper insights into how different carbon sources influence the metabolic responses of *C. militaris* upon light exposure, there were 30 such metabolic reactions uniquely associated with the sucrose culture upon light exposure. For example, r0192, associated with the CCM_00448 gene encoding sucrase (EC: 3.2.1.26). This enzyme is responsible for the hydrolysis of sucrose into its constituent monosaccharides, glucose and fructose. Importantly, it is a key enzyme in carbohydrate metabolism, playing essential roles in energy production and metabolic regulation. Moreover, we found r1283 to be involved in fatty acid metabolism, including long-chain-fatty-acid-CoA ligase (EC: 6.2.1.3; CCM_00448). This could potentially mitigate the toxicity resulting from the accumulation of excess glyoxylate generated during fatty acid metabolism [29]. Under glucose culture upon light exposure, there were 26 unique active metabolic reactions. For example, r0795 (EC: 2.3.2.2; EC: 3.4.19.13) associated with CCM_02065, CCM_02473, CCM_05577, CCM_05722, and CCM_09583 encoded for gamma-glutamyl transpeptidase to generate glutamate. It might be regulated and involved in the biosynthesis of cordycepin, serving as a precursor in the process [14,30].

### 3.5. Identified Reporter Metabolites of C. militaris

To identify the reporter metabolites of *C. militaris* cultures grown in glucose or sucrose upon light exposure, an integrative analysis was performed using the transcriptome data obtained from Thananusak et al. (2020) [13], and the retrofitted model was employed as a scaffold. As a result, 75 and 46 significant reporter metabolites in light–sucrose and light–glucose conditions were identified, respectively (Supplementary File S4; Table S4). Interestingly, the *i*PS1474 identified the top 20 reporter metabolites in response to light, which were involved in N-glycan biosynthesis, aminoacyl tRNA biosynthesis, cysteine and methionine metabolism, oxidative phosphorylation, phenylalanine, tyrosine and tryptophan biosynthesis, pyrimidine metabolism, lipid metabolism, and carotenoid biosynthesis, as shown in Table 2.

**Figure 7:**
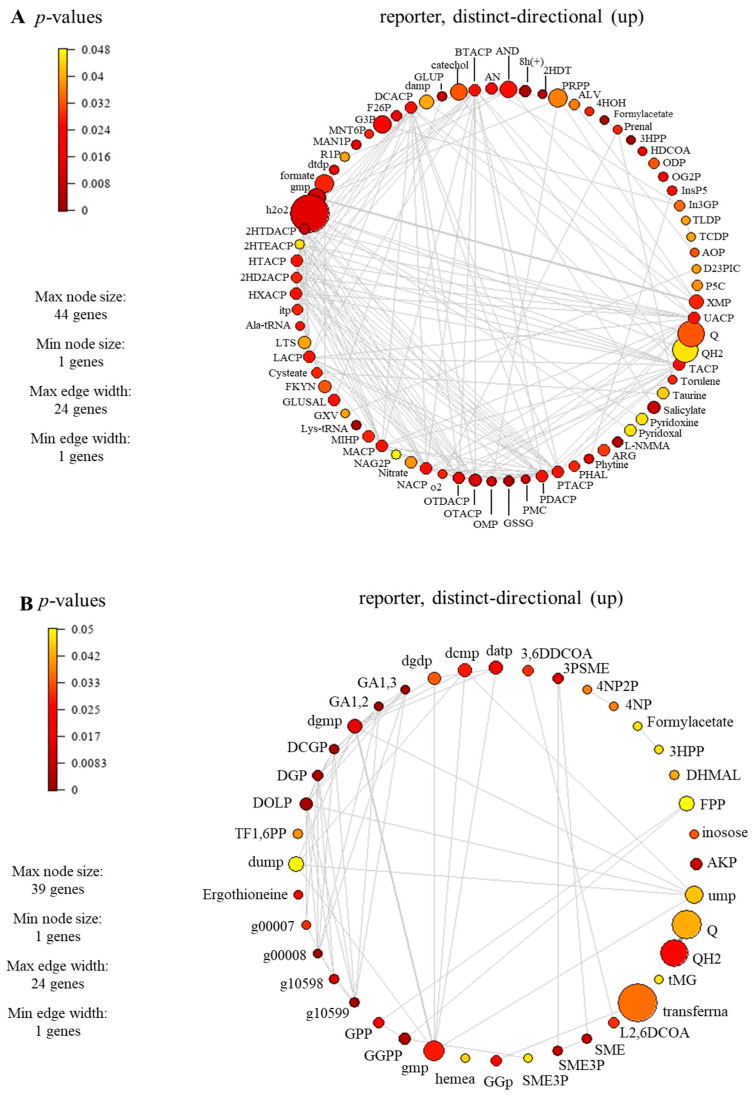
Subnetwork of reporter metabolites of *C. militaris* at different carbon sources under light exposure, (**A**) sucrose and (**B**) glucose. Full information of the subnetwork and full name of reporter metabolites can be seen in Supplementary File S4 (Tables S4 and S5).

The reporter metabolites participating in carotenoid biosynthesis, i.e., geranylgeranyl diphosphate (GGPP), geranyl diphosphate (GPP), torulene, and 2-trans,6-trans-farnesyl diphosphate (FPP), were identified, as seen in Table 2 and Supplementary File S4 (Table S4). For carotenoid-biosynthesis-associated genes, CCM_06728 encoding for torulene dioxygenase (EC: 1.13.11.59) and CCM_03203 encoding for farnesyl diphosphate synthase (EC: 2.5.1.10) were identified. These enzymes play a role in the degradation or conversion of torulene, which is indeed associated with carotenoid production. In the previous study, the influence of light affected the synthesis of carotenoid in *C. militaris* [31]. The synthesis of carotenoid is often regulated by light-dependent gene expression [13]. In the presence of light, genes responsible for carotenoid biosynthesis are activated, leading to an increase in the production of carotenoid. In addition, the significant reporter metabolites involved in the lipid metabolism were also found (e.g., butyryl-[acp], decanoyl-[acp], heptanoyl-[acp], hexanoyl-[acp], lauroyl-[acp], myristoyl-[acp], nonanoyl-[acp], pentadecanoyl-[acp], pentanoyl-[acp], tridecanoyl-[acp], undecanoyl-[acp], hexadecenoyl-[acp], heptadecanoyl-[acp], octadecenoyl-[acp], and 3-hydroxyoctadecanoyl-coa), as seen in Figure 7. The genes associated with these metabolites participated in the lipid biosynthetic pathway. For example, the genes encoding for secretory lipases, e.g., CCM_03046, CCM_04970, and CCM_09597. These enzymes facilitate the hydrolysis of neutral lipids (triacylglycerols) into free fatty acids. Subsequently, these free fatty acids diffuse through the membrane and are transported along with an acyl carrier protein to actively synthesize lipids within the fungal cell. It has been reported that the neutral lipids in lipid droplets are required during cordycepin accumulation in *C. militaris* [32].

## 4. Conclusions

The transcriptome-integrated GSMM approach was employed to extend the investigation of *C. militaris*’s metabolism under light conditions. Through the GIMME framework, the *i*PS1474-*ti*GSMM model was finally integrated with the transcriptome data, then provided a simulation that described the metabolic responses underlying the phenotypic observation of *C. militaris* under the particular light conditions. The *i*PS1474-*ti*GSMM-P50 under light–sucrose conditions outperformed to predict the metabolic fluxes of carotenoid biosynthesis. It could reveal the key metabolic route via the mevalonate pathway, which plays a crucial role in carotenoid biosynthesis. Beyond this, in silico gene knockout prediction and dissecting the complex interactions among transcription factors and target genes using this integrated modeling framework can be further studied.

## Figures and Tables

**Table 1:** Comparative metabolic characteristics of the GSMMs of *C. militaris*.

Model Characteristics	*i*NR1329 ^1^	This Study *i*PS1474
Total genes	1329	1474
Total metabolites	1171	1699
Total metabolic reactions	1821	1916
- Enzymatic reactions	1391	1412
- Non-enzymatic reactions	430	504
Spontaneous reactions	21	21
Transport reactions	271	336
Exchange reactions	137	146
Biomass reaction	1	1
Carotenoid biosynthesis	No	Yes

Note: Data were taken from ^1^ Raethong et al., 2020 [21].

**Table 2:** The *i*PS1474 model identified top 20 reporter metabolites in response to light.

Pathway	Reporter Metabolites	Sucrose Up-Directional *p*-Value	Glucose Up-Directional *p*-Value
N-Glycan biosynthesis	Dolichyl phosphate	-	0.001996
	Dolichyl-d-glucosyl phosphate	-	0.001996
	N-glycan (g10599)	-	0.001996
	D-glc-alpha-(1->2)	-	0.001996
	D-glc-alpha-(1->3)	-	0.001996
	N-glycan (g00008)	-	0.001996
	Dolichyl-beta-d-glucosyl phosphate	-	0.003992
	N-glycan (g10598)	-	0.005988
Aminoacyl tRNA biosynthesis	L-lysyl-tRNA(lys)	0.001996	-
Cysteine and methionine metabolism	Protein n (omega)-methyl-l-arginine	0.001996	-
Oxidative phosphorylation	8H (+) (energy metabolism)	0.003992	-
Phenylalanine, tyrosine and tryptophan biosynthesis	Shikimate3-phosphate	-	0.005988
	Shikimate	-	0.005988
	5-o-(1-carboxyvinyl)-3-phosphoshikimate	-	0.007984
Carotenoid biosynthesis	Geranylgeranyl diphosphate (GGPP)	-	0.005988
Pyrimidine metabolism	3-hydroxypropanoate	0.005988	-
	3-oxopropanoate	0.005988	-
Lipid metabolism	6,7-dihydropteridine	0.005988	-
	Oxidized glutathione	0.005988	-

The metabolite with a distinct up-directional *p*-value < 0.05.

## Data Availability

The model data presented in this study are openly available in GitHub (https://github.com/sysbiomics/Cordyceps_militaris-tiGSMM).

## Supplementary Materials

The following supporting information can be downloaded at: https://www.mdpi.com/article/10.3390/biology13030139/s1, Supplementary File S1: The transcriptome data with metabolic reactions of *i*PS1474 under light exposure, Table S1. Gene expression levels associated with metabolic reactions of *i*PS1474 (1412 genes); Supplementary File S2: The predicted fluxes of *i*PS1474 (FBA) and *i*PS1474-*ti*GSMM-P50 as well as gene expression of *C. militaris* under sucrose culture at light exposure, Table S2. The predicted fluxes of *i*PS1474 (FBA) and *i*PS1474-*ti*GSMM-P50 as well as gene expression of *C. militaris* under sucrose culture at light exposure; Supplementary File S3: Identified active light-exposed metabolic reactions predicted by *i*PS1474-*ti*GSMM-P50, Table S3. List of active metabolic reactions of *i*PS1474-*ti*GSMM-P50; Supplementary File S4: Identified reporter metabolites of *i*PS1474 in response to light, Table S4. List of reporter metabolites of *C. militaris* grown under sucrose culture, Table S5. List of reporter metabolites of *C. militaris* grown under glucose culture.

## References

[1] Cui J.D. (2015). Biotechnological production and applications of *Cordyceps militaris*, a valued traditional Chinese medicine. Crit. Rev. Biotechnol..

[2] Chan J., Barseghyan G., Asatiani M., Wasser S. (2015). Chemical composition and medicinal value of fruiting bodies and submerged cultured mycelia of caterpillar medicinal fungus *Cordyceps militaris* CBS-132098 (Ascomycetes). Int. J. Med. Mushrooms.

[3] Jędrejko K.J., Lazur J., Muszyńska B. (2021). *Cordyceps militaris*: An overview of its chemical constituents in relation to biological activity. Foods.

[4] Dong J., Lei C., Zheng X., Ai X., Wang Y., Wang Q. (2013). Light wavelengths regulate growth and active components of *Cordyceps militaris* fruit bodies. J. Food Biochem..

[5] Dong J., Wang S., Ai X., Yao L., Sun Z., Lei C., Wang Y., Wang Q. (2013). Composition and characterization of cordyxanthins from *Cordyceps militaris* fruit bodies. J. Funct. Foods.

[6] Dong Y., Jing T., Meng Q., Liu C., Hu S., Ma Y., Liu Y., Lu J., Cheng Y., Wang D. (2014). Studies on the antidiabetic activities of *Cordyceps militaris* extract in diet-streptozotocin-induced diabetic sprague-dawley rats. BioMed Res. Int..

[7] Liu C., Song J., Teng M., Zheng X., Li X., Tian Y., Pan M., Li Y., Lee R., Wang D. (2016). Antidiabetic and antinephritic activities of aqueous extract of *Cordyceps militaris* fruit body in diet-streptozotocin-induced diabetic sprague dawley rats. Oxidative Med. Cell. Longev..

[8] Rupa E.J., Li J.F., Arif M.H., Yaxi H., Puja A.M., Chan A.J., Hoang V.A., Kaliraj L., Yang D.C., Kang S.C. (2020). *Cordyceps militaris* fungus extracts-mediated nanoemulsion for improvement antioxidant, antimicrobial, and anti-inflammatory activities. Molecules.

[9] Tuli H.S., Sharma A., Sandhu S., Kashyap D. (2013). Cordycepin: A bioactive metabolite with therapeutic potential. Life Sci..

[10] Yu S.-H., Dubey N., Li W.-S., Liu M.-C., Chiang H.S., Leu S.-J., Shieh Y.-H., Tsai F.-C., Deng W.P. (2016). *Cordyceps militaris* treatment preserves renal function in type 2 diabetic nephropathy mice. PLoS ONE.

[11] Jiaojiao Z., Fen W., Kuanbo L., Qing L., Ying Y., Caihong D. (2018). Heat and light stresses affect metabolite production in the fruit body of the medicinal mushroom *Cordyceps militaris*. Appl. Microbiol. Biotechnol..

[12] Dong J.Z., Lei C., Ai X.R., Wang Y. (2012). Selenium enrichment on *Cordyceps militaris* link and analysis on its main active components. Appl. Biochem. Biotechnol..

[13] Thananusak R., Laoteng K., Raethong N., Zhang Y., Vongsangnak W. (2020). Metabolic responses of carotenoid and cordycepin biosynthetic pathways in *Cordyceps militaris* under light-programming exposure through genome-wide transcriptional analysis. Biology.

[14] Raethong N., Wang H., Nielsen J., Vongsangnak W. (2020). Optimizing cultivation of *Cordyceps militaris* for fast growth and cordycepin overproduction using rational design of synthetic media. Comput. Struct. Biotechnol. J..

[15] Cheawchanlertfa P., Chitcharoen S., Raethong N., Liu Q., Chumnanpuen P., Soommat P., Song Y., Koffas M., Laoteng K., Vongsangnak W. (2022). Enhancing Genome-Scale Model by Integrative Exometabolome and Transcriptome: Unveiling carbon assimilation towards sphingolipid biosynthetic capability of *Cordyceps militaris*. J. Fungi.

[16] Becker S.A., Palsson B.O. (2008). Context-specific metabolic networks are consistent with experiments. PLoS Comput. Biol..

[17] Zur H., Ruppin E., Shlomi T. (2010). iMAT: An integrative metabolic analysis tool. Bioinformatics.

[18] Robaina Estévez S., Nikoloski Z. (2015). Context-specific metabolic model extraction based on regularized least squares optimization. PLoS ONE.

[19] Casini I., McCubbin T., Esquivel-Elizondo S., Luque G.G., Evseeva D., Fink C., Beblawy S., Youngblut N.D., Aristilde L., Huson D.H. (2023). An integrated systems biology approach reveals differences in formate metabolism in the genus *Methanothermobacter*. iScience.

[20] Siriwach R., Matsuda F., Yano K., Hirai M.Y. (2020). Drought stress responses in context-specific genome-scale metabolic models of *Arabidopsis thaliana*. Metabolites.

[21] Kamsen R., Kalapanulak S., Chiewchankaset P., Saithong T. (2021). Transcriptome integrated metabolic modeling of carbon assimilation underlying storage root development in cassava. Sci. Rep..

[22] Kanehisa M., Sato Y., Kawashima M., Furumichi M., Tanabe M. (2016). KEGG as a reference resource for gene and protein annotation. Nucleic Acids Res..

[23] Caspi R., Billington R., Fulcher C.A., Keseler I.M., Kothari A., Krummenacker M., Latendresse M., Midford P.E., Ong Q., Ong W.K. (2018). The MetaCyc database of metabolic pathways and enzymes. Nucleic Acids Res..

[24] Wang H., Marcišauskas S., Sánchez B.J., Domenzain I., Hermansson D., Agren R., Nielsen J., Kerkhoven E.J. (2018). RAVEN 2.0: A versatile toolbox for metabolic network reconstruction and a case study on *Streptomyces coelicolor*. PLoS Comput. Biol..

[25] Heirendt L., Arreckx S., Pfau T., Mendoza S.N., Richelle A., Heinken A., Haraldsdóttir H.S., Wachowiak J., Keating S.M., Vlasov V. (2019). Creation and analysis of biochemical constraint-based models using the COBRA Toolbox v.3.0. Nat. Protoc..

[26] Väremo L., Nielsen J., Nookaew I. (2013). Enriching the gene set analysis of genome-wide data by incorporating directionality of gene expression and combining statistical hypotheses and methods. Nucleic Acids Res..

[27] Park J.P., Kim S.W., Hwang H.J., Yun J.W. (2001). Optimization of submerged culture conditions for the mycelial growth and exo-biopolymer production by *Cordyceps militaris*. Lett. Appl. Microbiol..

[28] Suparmin A., Kato T., Dohra H., Park E.Y. (2017). Insight into cordycepin biosynthesis of *Cordyceps militaris*: Comparison between a liquid surface culture and a submerged culture through transcriptomic analysis. PLoS ONE.

[29] Puckett S., Trujillo C., Wang Z., Eoh H., Ioerger T.R., Krieger I., Sacchettini J., Schnappinger D., Rhee K.Y., Ehrt S. (2017). Glyoxylate detoxification is an essential function of malate synthase required for carbon assimilation in *Mycobacterium tuberculosis*. Proc. Natl. Acad. Sci. USA.

[30] Oh J., Yoon D.-H., Shrestha B., Choi H.-K., Sung G.H. (2019). Metabolomic profiling reveals enrichment of cordycepin in senescence process of *Cordyceps militaris* fruit bodies. J. Microbiol..

[31] Yang T., Guo M., Yang H., Guo S., Dong C. (2016). The blue-light receptor CmWC-1 mediates fruit body development and secondary metabolism in *Cordyceps militaris*. Appl. Microbiol. Biotechnol..

[32] Qin P., Wang Z., Lu D., Kang H., Li G., Guo R., Zhao Y., Han R., Ji B., Zeng Y. (2019). Neutral lipid content in lipid droplets: Potential biomarker of cordycepin accumulation in cordycepin-producing fungi. Molecules.

